# Phase 1 trial of the antiangiogenic peptide ATN-161 (Ac-PHSCN-NH_2_), a beta integrin antagonist, in patients with solid tumours

**DOI:** 10.1038/sj.bjc.6603171

**Published:** 2006-05-16

**Authors:** M E Cianfrocca, K A Kimmel, J Gallo, T Cardoso, M M Brown, G Hudes, N Lewis, L Weiner, G N Lam, S C Brown, D E Shaw, A P Mazar, R B Cohen

**Affiliations:** 1 Fox Chase Cancer Center, 333 Cottman Avenue, Philadelphia, PA 19111, USA; 2 Attenuon, LLC, San Diego, CA, USA; 3 DE Shaw Research and Development, LLC, New York, NY USA; 4 MicroConstants Inc., San Diego, CA, USA

**Keywords:** ATN-161, *α*5*β*1 integrin, *α*v*β*3 integrin, phase 1 trial, angiogenesis

## Abstract

To evaluate the toxicity, pharmacological and biological properties of ATN-161, a five –amino-acid peptide derived from the synergy region of fibronectin, adult patients with advanced solid tumours were enrolled in eight sequential dose cohorts (0.1–16 mg kg^−1^), receiving ATN-161 administered as a 10-min infusion thrice weekly. Pharmacokinetic sampling of blood and urine over 7 h was performed on Day 1. Twenty-six patients received from 1 to 14 4-week cycles of treatment. The total number of cycles administered to all patients was 86, without dose-limiting toxicities. At dose levels above 0.5 mg kg^−1^, mean total clearance and volume of distribution showed dose-independent pharmacokinetics (PKs). At 8.0 and 16.0 mg kg^−1^, clearance of ATN-161 was reduced, suggesting saturable PKs. Dose escalation was halted at 16 mg kg^−1^ when drug exposure (area under the curve) exceeded that associated with efficacy in animal models. There were no objective responses. Six patients received more than four cycles of treatment (>112 days). Three patients received 10 or more cycles (⩾280 days). ATN-161 was well tolerated at all dose levels. Approximately, 1/3 of the patients in the study manifested prolonged stable disease. These findings suggest that ATN-161 should be investigated further as an antiangiogenic and antimetastatic cancer agent alone or with chemotherapy.

Angiogenesis is a requirement for the growth of nearly all tumours and most metastases (Folkman, 1995; Carmeliet and Jain, 2000). Regulatory molecules influencing endothelial cell behaviour include: (1) circulating cytokines and growth factors, (2) extracellular matrix (ECM) components and (3) membrane-bound proteins. Antiangiogenic therapy could theoretically target any step in the angiogenic process (Folkman, 1995; Harris, 1997).

Fibronectin is a component of the ECM that is also found at high concentrations in plasma. Fibronectin interacts with a variety of cell types including endothelial cells and regulates adhesion, growth and migration through binding to the integrin family of transmembrane proteins. Integrins are heterodimeric cell surface receptors comprised of *α* and *β* subunits that mediate endothelial cell proliferation and migration, both crucial features of neovessel establishment (Brooks *et al*, 1994a, 1994b; Brooks, 1996; Mitjans *et al*, 2000). The central cell-binding domain of fibronectin contains the RGD recognition sequence required for binding to *α*_5_*β*_1_ integrin (Pierschbacher and Ruoslahti, 1984) and the PHSRN synergy sequence that increases the affinity and specificity of RGD-mediated binding. (Aota *et al*, 1994; Mould *et al*, 1997) An unregulated invasive response to the PHSRN synergy sequence may contribute significantly to the growth, survival and metastasis of established tumours.(Livant *et al*, 2000a) The role of the PHSRN sequence in promoting tumour invasion and angiogenesis makes it an appealing target for cancer therapy.

ATN-161 is a noncompetitive inhibitor of the fibronectin PHSRN sequence, in which a cysteine residue has been substituted for arginine along with peptide acetylation and amidation in order to yield a product with acceptable pharmaceutical properties (Ac-PHSCN-NH_2_). Unlike other integrin antagonists ATN-161 does not block integrin-dependent adhesion, but may inhibit integrin-dependent signalling as part of its mechanism of action (Plunkett and Mazar, 2002; Plunkett *et al*, 2002). Recent studies show that ATN-161 binds exclusively to integrin beta subunits (Donate *et al*, 2003). Thus, ATN-161 may inhibit the function of several integrins implicated in tumour angiogenesis and metastasis. Disulphide interchange has been proposed to mediate integrin activation (Yan and Smith, 2000); we hypothesise that the free cysteine thiol in ATN-161 blocks this interchange by forming a disulphide with the integrin target, thereby suppressing integrin function.

*In vitro*, ATN-161 inhibited PHSRN-induced basement membrane invasion of human (DU145) and rat (MLL) prostate cancer cell lines (Livant *et al*, 2000b). *In vivo*, systemic administration of 5 mg kg^−1^ ATN-161 (five injections over 16 days) to Copenhagen rats markedly reduced the growth of primary MLL tumours. Furthermore, immunostaining of tumour sections from treated and untreated rats suggested that blood vessel density in tumour tissue from ATN-161-treated animals was eight- to 10-fold lower on Day 16 than in tumour tissue from untreated animals. ATN-161 also inhibited the ability of MLL tumour cells to metastasise. Attempts to show induction of apoptosis in MLL cells by ATN-161 were unsuccessful, suggesting that the inhibitory effects of ATN-161 on primary tumour growth and metastasis formation were the result of inhibition of new blood vessel growth rather than a direct effect on tumour cells. We have also generated preclinical data showing additive effects with diverse chemotherapy agents (Plunkett *et al*, 2002; Plunkett *et al*, 2003; Stoeltzing *et al*, 2003). ATN-161 was not immunogenic in animal studies.

In preclinical efficacy models ATN-161 exhibited a U-shaped (inverted bell shape) dose–response curve. These preclinical animal models included assessment of the effects of ATN-161 on tumour growth, metastasis, angiogenesis, tumour perfusion and circulating endothelial progenitor cells (CEPs) (Donate *et al*, 2003). Preclinical toxicology studies showed no consistent evidence of ATN-161 toxicity in rats or primates except at extremely high, supratherapeutic doses. We designed the phase 1 trial to evaluate a dose range in human beings (using well-established rules for interspecies dose conversion (Freireich *et al*, 1966)) that would cover adequately the broad trough of the U-shaped dose–response curve.

This phase 1 clinical trial is the first study of this novel peptide in humans. The thrice-weekly i.v. infusion schedule was chosen because in murine studies frequent dosing was more efficacious than intermittent dosing with little difference between dosing daily and three times per week. The study also aimed to describe any dose-limiting toxicities (DLTs) of ATN-161 and to verify the absence of a maximum tolerated dose (MTD) in the chosen dose range. Secondary objectives of the trial were to assess the pharmacokinetics (PKs) of ATN-161 and to describe any preliminary evidence of antitumour activity.

## PATIENTS AND METHODS

### Patient selection

Patients were eligible if they had a histologically or cytologically confirmed nonhaematologic malignancy that was either unresponsive to standard therapies or for which there was no known effective therapy. Patients with measurable and nonmeasurable disease were eligible. Other eligibility criteria included the following: Eastern Cooperative Oncology Group performance status (ECOG PS) ⩽2 (Oken *et al*, 1982), age ⩾18 years, adequate haematologic variables (absolute neutrophil count ⩾1500 cells mm^−3^, platelet count ⩾100 000 cells mm^−3^), adequate hepatic function (total bilirubin ⩽1.8 mg dl^−1^ and transaminase levels ⩽2.5 times the institutional upper limits of normal (ULN) (⩽5 times ULN for patients with liver metastases)), and adequate renal function (serum creatinine ⩽1.5 times the institutional ULN). Prior chemo- or radiation therapy or immunotherapy was allowed more than 4 weeks before Day 1 of study treatment (6 weeks for nitrosoureas, mitomycin-C and liposomal doxorubicin). Treatment-related toxicities of any prior therapies, including surgery, must have resolved to Grade 1 or less prior to Day 1 of study treatment. Exclusion criteria included the following: pregnant or nursing women; men who had not had a vasectomy or were not using birth control; patients with an active, unresolved infection or who had received antibiotics within 7 days of the start of study treatment; patients with carcinomatous meningitis or untreated/uncontrolled metastatic parenchymal brain disease. Patients with a prior history of parenchymal brain disease, which had been controlled with appropriate therapy, were eligible if at least 8 weeks had passed since completion of therapy, and the patient was asymptomatic and off corticosteroids. The Fox Chase Cancer Center Institutional Review Board (IRB) approved the study protocol and all patients signed an IRB-approved written informed consent prior to study procedures and treatment.

### Treatment plan and study design

ATN-161 was supplied as a sterile-filled, lyophilised powder in glass vials (5 ml), which contained 100 mg ATN-161 for injection, 100 mg glycine and 50 mmol sodium citrate (pH adjusted to pH 5.0. using NaOH prior to lyophilisation). Each vial of ATN-161 for injection was reconstituted to 2 ml using sterile water for injection, and then diluted in i.v. bags containing 50 or 100 ml of sterile normal saline USP.

ATN-161 was administered as a 10-min infusion, three times per week. Sequential cohorts of three patients were enrolled to eight different dose levels (0.1, 0.25, 0.5, 1.0, 2.0, 4.0, 8.0 and 16.0 mg kg^−1^). All patients in a given dose level were evaluated for at least 28 days before patients could be treated at the next higher dose level. Patients receiving therapy for <1 cycle (4 weeks) were to be replaced unless they had experienced a DLT. Toxicity was evaluated according to the NCI Common Terminology Criteria (CTC) Version 2.0. Dose-limiting toxicities were defined as any of the following treatment-related events occurring during the first cycle of therapy: Grade 4 neutropenia or febrile neutropenia, any other Grade 3 or greater nonhaematologic toxicity, or any toxicity requiring ATN-161 to be held for more than 1 week (i.e., three consecutive missed doses). In the event of DLT, dosing was to be held until recovery to at least Grade 1 toxicity and a new dosing cycle for that patient was to be initiated at the investigator's discretion at 50% of the previous dose. If recovery did not occur within 1 week (three missed doses), however, study treatment was to be discontinued. In the event of Grade 2 toxicity involving vital organs (hepatic, pulmonary, renal, neurologic, gastrointestinal or cardiotoxicity), the dose of ATN-161 was to be held until recovery to Grade 0–1 before a new cycle was initiated at 100% dosing. If the Grade 2 toxicity recurred, dosing was to be held until recovery to Grade 0–1 and the next cycle would resume at 50%. Occurrence of DLT at a given dose level would require further enrolment of up to six patients at that dose level to determine if the MTD had been exceeded. The MTD was defined as one dose level below which ⩾2/6 patients experienced a DLT. Treatment could continue until progressive disease, or one of the following: unacceptable toxicity, intercurrent illness, patient declined further treatment, or physician decision in view of a patient's other medical conditions.

### Patient evaluation

Pretreatment evaluations were performed within 14 days prior to therapy, except for X-rays and scans, which were performed within 28 days before therapy. Initial patient assessments included a complete history and physical examination, including neurological examination, weight, vital signs, ECOG PS, chest X-ray, electrocardiogram, complete blood cell count with differential and platelets, lactate dehydrogenase, glucose, electrolytes, albumin, blood urea nitrogen, creatinine, aspartate aminotransferase (serum glutamic oxaloacetic transaminase), alanine aminotransferase (serum glutamic pyruvic transaminase), alkaline phosphatase, total bilirubin, magnesium, calcium, phosphorus, triglycerides, fibrinogen, PT and partial thromboplastin time, and a complete urinalysis. For women of childbearing potential, a negative serum pregnancy test was documented within 72 h prior to the start of treatment. Tumour assessments were by physical examination, CT scan and/or MRI, as appropriate.

During treatment, patients were evaluated monthly with physical examinations including toxicity assessment using the CTC, ECOG PS, serum chemistry, haematology and coagulation testing. Tumour burden was reassessed every two cycles (8 weeks) by physical exam or radiological methods as appropriate using RECIST (Therasse *et al*, 2000).

### Pharmacokinetic sampling and analysis

Blood samples for PK analysis were collected in EDTA-coated tubes on Day 1 for all patients preinfusion, at the end of infusion, and at 5, 10, 15, 30, 45, 60, 90, 120, 150, 180, 240, 400, 460 and 520 min after infusion. Samples were immediately centrifuged, and the plasma was separated and stored at −20°C prior to analysis. To determine renal clearance (CLr) of ATN-161, urine samples were also collected on Day 1 over the same 7-h postdose period. Plasma and urine were analysed for ATN-161 by HPLC-MS/MS assays. The measured concentration was the sum of free ATN-161, dimerised ATN-161, and protein-bound ATN-161 after incubation with a *tris*(carboxyethyl)phosphine solution.

Pharmacokinetic data were modelled using a noncompartmental approach to estimate individual subject values for the following parameters: maximum observed concentration (*C*_max_), time of the maximum observed concentration (*T*_max_), volume of distribution at steady state (VSS), terminal elimination half-life (*t*_1/2_), total systemic clearance (CL), area under the plasma concentration-time curve (AUC), and CLr. For these parameters the mean and s.d. coefficient of variation were calculated.

### Statistical analysis

No inferential analyses were performed. Data are presented in a descriptive fashion. Treatment-emergent adverse events were translated from investigator terms to MedDRA terminology and summarised within and across dose levels for all patients who received at least one dose of ATN-161. Treatment administration is described for all patients, including drug/dose administration, number of cycles, dose modifications or delays and duration of therapy.

Descriptive PKs parameters were determined by standard model independent methods for infusion administration (Perrier and Gibaldi, 1982) from plasma concentrations in each individual patient. Plasma and urine concentrations were rounded to the nearest 
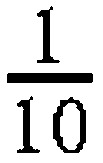
 ng ml^−1^ before the calculations. Plasma samples with concentrations below the quantifiable limit of 50.0 ng ml^−1^ (BQL) were assigned values of zero for generation of mean and s.d. plasma concentrations. For PKs calculations, BQL was treated as zero up to the first quantifiable plasma concentration and then treated as an empty value thereafter. Urine samples reported as BQL (⩽50.0 ng ml^−1^) were treated as zero for all calculations. Actual patient blood collection sampling times relative to the start of infusion were used for PKs calculations; however, scheduled collection times were used for urinary excretion data and graphical presentations of all data.

## RESULTS

### Patient characteristics

As shown in Table 1, 26 adult patients were enrolled into the study from January 2003 to October 2004 at the Fox Chase Cancer Center. The median age for all patients was 64 years (range 26–91 years), and there were 15 males and 11 females. All patients had pathological diagnoses of cancer as indicated. Most patients were heavily pretreated but three patients had received no prior chemotherapy (although each had received radiation or biologic therapy). The majority of patients had an ECOG PS of 1. Three patients were enrolled in each cohort except for the 0.25 and 1.0 mg kg^−1^ cohorts, into which an additional patient was enrolled as a replacement for a patient who withdrew prematurely from the study (for reasons other than toxicity).

### Toxicity

Table 2 shows the eight dose levels for the study and all adverse events at each dose level in any course of treatment. Few adverse events were considered treatment related and none of these events was considered both serious and treatment associated at any of the dose levels tested. All of the treatment-related adverse events were Grade 2 or less and no DLTs occurred. There was no indication that the incidence or severity of adverse events increased at higher doses or that side effects emerged or became worse with continued chronic dosing. A MTD was not reached at 16 mg kg^−1^, which was therefore designated as the maximum administered dose. One patient at the 8 mg kg^−1^ dose level died suddenly during Cycle 2. The event was felt due to underlying cardiac disease and not to protocol therapy. No autopsy was performed, however, and therefore a precise cause of death was not determined.

### Pharmacokinetics

Pharmacokinetics data were obtained from all patients treated with ATN-161. At the lowest dose level of 0.1 mg kg^−1^, ATN-161 was detected only at the earliest plasma sampling time points (up to 15 min postinfusion); therefore, data from that dose level are not included in the PK summary in Table 3. Pharmacokinetic data at doses of 0.25 and 0.5 mg kg^−1^ showed considerable interpatient variability, in part due to undetectable plasma concentrations at later time sampling points. At dose levels 1.0, 2.0 and 4.0 mg kg^−1^, PK parameters appear to be dose independent. At the 8 and 16 mg kg^−1^ dose levels, *C*_max_ and AUCs were increased, and total clearance was reduced, suggesting saturable elimination. The observed half-life was 3.2–5.0 h. At doses of ATN-161 above 0.5 mg kg^−1^, *t*_1/2_ values showed little variability. There was no serological evidence for an antibody immune response against ATN-161.

Urinary excretion of ATN-161 accounted for approximately 2% of the administered ATN-161 dose in the majority of the patients (20 of 26) and between 20 and 50% of the dose in the remaining six patients. This dichotomy in excretion percentages was not associated with dose level. In almost all patients (23 of 26), the majority of the dose was excreted during the first 2 h following dose administration. Mean CLr of ATN-161 ranged from 18.2 to 52.4 ml min^−1^ across all dose groups.

### Clinical response

Table 4 shows the duration of treatment and clinical response for all patients treated. No patient in the study had an objective response as defined by RECIST. The median number of cycles received was 2, which corresponded to 8 weeks of treatment. Treatment duration ranged from <1 to 14 cycles. There was no apparent correlation between clinical benefit and dose level nor was there a clear association between length of time on study and dose level. Among the 26 treated patients, six (23%) experienced stable disease of >4 months and three of those patients received 10 or more cycles (Table 4). Four patients with prolonged stable disease had well-documented progressive disease prior to study entry (one case each of adenoid cystic, renal cell, prostate and ovarian cancer).

## DISCUSSION

We report here the results of a first-in-humans phase 1, dose escalation, PKs study of thrice weekly i.v. ATN-161 in patients with advanced cancer. Patients were treated at doses ranging from 0.1 to 16 mg kg^−1^. Our study demonstrates that treatment with ATN-161 was without significant toxicity in the assessed dose range; we have defined 16 mg kg^−1^ as the maximum administered dose. There were no objective clinical responses.

Pharmacokinetics data showed rapid clearance from plasma and a Vss indicating high tissue distribution. Despite a very short half-life, ATN-161 appeared to have a durable effect in suppressing tumour growth in preclinical model systems such that it only needs to be administered intermittently (rather than continuously). We therefore hypothesise either that the peptide is retained on its receptor for a period of time (consistent with the hypothesis that ATN-161 forms a disulphide bond with its target integrin) or that ATN-161 otherwise elicits a long-lived response. Studies are currently underway in preclinical models to evaluate these hypotheses. ATN-161 was not immunogenic in humans, confirming the preclinical animal observations, and indicating that immunogenicity does not explain the short half-life.

We did not determine a traditional MTD for ATN-161. The dose range for the phase 1 trial was chosen to bracket the most active portion (trough) of the U, converting the active dose range from mouse to man using well-established rules of interspecies dose conversion (Freireich *et al*, 1966). Given the shape of the preclinical dose–response curve there would, in fact, be no purpose in aiming for or achieving a traditional MTD. The absence of an MTD, of course, makes dose selection for phase 2 studies challenging. We believe the efficacious dose range was probably covered by the doses evaluated in the phase 1 study; each of these doses was safe. In future studies in phase 2 patient populations that are more homogeneous for disease, stage, and prior treatment history, we will assess biomarkers such as CEPs (Bertolini *et al*, 2003), and dce-MRI (Galbraith *et al*, 2003; Liu *et al*, 2005) to refine dose selection. Preclinical studies of ATN-161, for example, suggest that at least one of these biomarkers also exhibits a U-shape in the response to ATN-161. (Hensley and Mazar, 2003)

U-shaped dose response curves (low-dose stimulation and high-dose inhibition) occur in other systems, including studies of chemotherapeutic agents (Calabrese and Baldwin, 2002, 2003). At least one set of empiric observations in the angiogenesis field suggests that interferon alpha at a low dose rather than a maximal tolerated dose has the most potent clinical effects (Drolet *et al*, 1999; Slaton *et al*, 1999). Similar dose–response curves have been described for immunocytokines (Kopp *et al*, 1993; Talmadge, 1998; Curnis *et al*, 2005). Phase 1 clinical trials experts have suggested that an MTD-seeking approach may not be appropriate for antineoplastic agents with mechanisms of action that are distinct from conventional cytotoxic drugs (Parulekar and Eisenhauer, 2004).

ATN-161 is the first inhibitor of the PHSRN synergy sequence to be evaluated in a clinical trial. There are published studies of other agents that target integrins for cancer therapy, including MEDI-532 (Vitaxin™, an anti-*α*v*β*_3_ antibody) (Gutheil *et al*, 2000) and cilengitide (EMD 121974, a cyclised pentapeptide that is a potent and selective antagonist for integrins *α*v*β*3 and *α*v*β*5) (Eskens *et al*, 2000). As we observed with ATN-161, integrin antagonists with diverse molecular structures are safe and potentially active without evidence for dose-limiting toxicity. Interestingly, there were no objective responses to any of these agents, but there were descriptions of prolonged stable disease, especially in patients with renal cell cancer (Eskens *et al*, 2000).

The safety and tolerability of ATN-161 in this study and preliminary evidence of possible biologic effect (prolonged stable disease) suggest that further clinical studies of this agent are warranted. Similar to the clinical development of other agents such as bevacizumab (Hurwitz *et al*, 2004; Wakelee and Schiller, 2005; Ahmed *et al*, 2004; Rini *et al*, 2005; Sledge, 2005), the best approach for phase 2 studies will be combinations of ATN-161 with chemotherapy rather than development as a single agent (Plunkett *et al*, 2002, 2003; Stoeltzing *et al*, 2003).

## Figures and Tables

**Table 1:** Patient characteristics (*N*=26)

*Sex (N)*	
Men	15
Women	11
	
*Age, years*	
Median	64
Min–max	(26–91)
	
*Number of prior chemotherapy regimens*	
0	3^a^
1	7
2	4
⩾3	12
	
*ECOG performance status*	
0	6
1	15
2	5
	
*Tumour type/tumour location (N)*	
Prostate	4
Hepatocellular	3
Colon	2
Renal cell	2
Other, one each^b^	15

a Adenoid cystic cancer treated with surgery and radiation, renal cell cancers treated with immunotherapy and anti-EGFR monoclonal antibody.

b Adenoid cystic carcinoma of hard palate, ameloblastoma, breast, carcinoid neuroendocrine tumour, oesophageal, liposarcoma, Merkel cell, non-small-cell lung cancer, osteosarcoma, ovarian (granulosa cell), pancreatic, primitive neuroectodermal tumour (kidney), pseudomyxoma peritonei, thyroid, unknown primary.

**Table 2:** ATN-161-associated^a^ adverse events by dose level

**MedDRA preferred term**	**Number of patients**
*0.5 mg kg* ^*−1*^	1
Muscle cramps	1
Sweating increased	1
	
*1.0 mg kg* ^*−1*^	1
Dry mouth	1
Cellulitis	1
Pain in limb	1
Paresthesia	1
Phlebitis not otherwise specified (NOS)	1
	
*2.0 mg kg* ^*−1*^	2
Oral candidiasis	1
Anemia NOS	1
Hypotension NOS	1
	
*4.0 mg kg* ^*−1*^	2
Nausea	2
Constipation	1
Fatigue	1
	
*8.0 mg kg* ^*−1*^	1
Nausea	1
	
*16.0 mg kg* ^*−1*^	1
Hypotension NOS	1

a Possible, probable or definite relationship to study medication according to the investigator.

**Table 3:** Pharmacokinetic properties of ATN-161 in patients*

	**Dose (mg kg^−1^)**							
**Parameter**	**0.1**	**0.25**	**0.5**	**1**	**2**	**4**	**8**	**16**
Dose level	I	II	III	IV	V	VI	VII	VIII
*C*_max_ (ng ml^−1^)	236.0	1119.9	5785.5	3800.5	15730.8	5476.3	34414.1	72673.1
AUC(0-7) (ng h ml^−1^)	ND	3415.1	3608.2	1446.3	3317.0	3712.8	34216.9	54927.5
AUC(0-24) (ng h^−1^ ml^−1^)	ND	5108.5	4302.6	1759.0	3672.7	4726.3	40774.2	74203.3
AUC(0-inf) (ng h^−1^ ml^−1^)	ND	5394.6	4382.6	1781.3	3693.9	4915.1	41045.1	78754.8
CL (l h^−1^ kg^−1^)	ND	0.08	0.17	0.61	0.69	0.87	0.42	0.35
CLr (l h^−1^ kg^−1^)	ND	0.024	0.031	0.014	0.019	0.026	0.030	0.033
CLr (ml min^−1^)	ND	23.33	52.38	18.16	23.25	29.61	35.73	48.09
*V*_d_ (l kg^−1^)	ND	0.48	0.87	3.38	3.38	5.59	2.14	2.36
Vss (l kg^−1^)	ND	0.33	0.47	2.30	1.93	3.82	1.32	1.61
MRT(INF) (h)	ND	5.5	3.3	3.8	2.5	4.8	3.4	5.3
Urinary excretion (%)	2.46	13.79	25.31	1.82	2.35	2.36	17.49	15.26
*T*_1/2_^a^ (h)	ND	4.4	3.2	3.9	3.5	4.3	3.3	5.0

* All values represent the mean (s.d.) except for *T*_1/2_.

ND, not determined; CL, systemic clearance; CLr, renal clearance; Vd, volume of distribution; *V*_dss_, volume of distribution at steady-state; MRT(INF), mean residence time; urinary excretion, urinary excretion between 0 and 7 h as a percent of dose.

a Expressed as harmonic mean.

**Table 4:** Number of cycles administered

**Number of cycles**	**Dose level (mg kg^−1^)**	**Number of patients**
⩽2	0.1–16.0	16
>2–⩽4	0.1–1.0	4
4.5	0.5	1: prostate
5	2.0	1: pseudomyxoma peritonei
6	16.0	1: ameloblastoma
10	0.1	1: ovarian granulosa cell tumour
11	2.0	1: renal cell
14	1.0	1: adenoid cystic of hard palate
